# Single-cell analysis reveals specific neuronal transition during mouse corticogenesis

**DOI:** 10.3389/fcell.2023.1209320

**Published:** 2023-11-06

**Authors:** Ziheng Zhou, Yueyang Pan, Si Zhou, Shuguang Wang, Dengwei Zhang, Ye Cao, Xiaosen Jiang, Jie Li, Linnan Zhu, Lijian Zhao, Shen Gu, Ge Lin, Zirui Dong, Hai-Xi Sun

**Affiliations:** ^1^ Department of Obstetrics and Gynaecology, The Chinese University of Hong Kong, Shatin, Hong Kong SAR, China; ^2^ College of Life Sciences, University of Chinese Academy of Sciences, Beijing, China; ^3^ Shenzhen Research Institute, The Chinese University of Hong Kong, Shenzhen, China; ^4^ School of Biomedical Sciences, Faculty of Medicine, The Chinese University of Hong Kong, Shatin, Hong Kong SAR, China; ^5^ Bioinformatics Department, School of Life Sciences and Technology, Tongji University, Shanghai, China; ^6^ Biomedical Pioneering Innovation Center (BIOPIC), Academy for Advanced Interdisciplinary Studies, School of Life Sciences, Peking University, Beijing, China; ^7^ Medical Technology College, Hebei Medical University, Shijiazhuang, Hebei, China

**Keywords:** single-cell RNA sequencing, cerebral cortex development, neuronal transition, neuronal migration, deep-layer neurons, upper-layer neurons

## Abstract

**Background:** Currently, the mechanism(s) underlying corticogenesis is still under characterization.

**Methods:** We curated the most comprehensive single-cell RNA-seq (scRNA-seq) datasets from mouse and human fetal cortexes for data analysis and confirmed the findings with co-immunostaining experiments.

**Results:** By analyzing the developmental trajectories with scRNA-seq datasets in mice, we identified a specific developmental sub-path contributed by a cell-population expressing both deep- and upper-layer neurons (DLNs and ULNs) specific markers, which occurred on E13.5 but was absent in adults. In this cell-population, the percentages of cells expressing DLN and ULN markers decreased and increased, respectively, during the development suggesting direct neuronal transition (namely D-T-U). Whilst genes significantly highly/uniquely expressed in D-T-U cell population were significantly enriched in PTN/MDK signaling pathways related to cell migration. Both findings were further confirmed by co-immunostaining with DLNs, ULNs and D-T-U specific markers across different timepoints. Furthermore, six genes (co-expressed with D-T-U specific markers in mice) showing a potential opposite temporal expression between human and mouse during fetal cortical development were associated with neuronal migration and cognitive functions. In adult prefrontal cortexes (PFC), D-T-U specific genes were expressed in neurons from different layers between humans and mice.

**Conclusion:** Our study characterizes a specific cell population D-T-U showing direct DLNs to ULNs neuronal transition and migration during fetal cortical development in mice. It is potentially associated with the difference of cortical development in humans and mice.

## Background

Development of cerebral cortex involves multiple biological processes that lead to the formation of functional neural networks. It includes cell proliferation, differentiation, cell fate commitment, migration, and programmed cell death during the development. In mammals, cortical progenitors rapidly divide into a wide variety of cell types including neurons and non-neuron cells, giving rise to the formation of six neocortical layers (Greig et al., 2013). Precise investigation on the spatial and temporal cellular compositions and regulation of gene expressions is important to understand the underly mechanisms during development of mammalian nervous system, and the differences among different species.

Mouse models are commonly used to manipulate human cognitive abilities and susceptibility to disease as both humans and mice have six histologically distinct layers. By the analysis of gene-knockout mice with/without lineage tracing technologies, studies demonstrate the molecular mechanisms of neuronal subtype transitions during corticogenesis: the induction of *Foxg1* at progressively later stages during development (E14.5–E16.5) showed that UL progenitors are unable to bypass DL competence for their production even at the latest period of corticogenesis (Toma and Hanashima, 2015). In addition, the transition from producing DLNs to ULNs is regulated by signals propagated from postmitotic DLNs, terminating DLN production through negative feedback (Toma and Hanashima, 2015). Although it is well known that ULNs are generated from UL progenitors, the question remains unclear whether ULNs can be generated by direct transition from DLNs. To answer this question, a study applying bulk RNA-seq from Deep Layer Neurons (DLNs) and Upper Layer Neurons (ULNs) in early, mid, and late developmental stages in mice identified a cluster of ULN-like transcripts in DLNs (with *Mpped1* as a marker gene). An evolving model of progressive restriction of cell fate competence through inherited transcriptional identities was proposed but the study only focused on the expression pattern of gene *Myt1* (Heavner et al., 2020). In addition, analyses show motifs for the transcriptional factors (TFs) associated with neurogenesis and neuronal differentiation, were enriched in both lineages of callosal projection neurons (CPNs) and corticofugal projection neurons (CFuPNs) in mice, suggesting that fates diverge during the acquisition of post-mitotic neuronal identity (Di Bella et al., 2021). Recently, with the advancement of single-cell RNA sequencing (scRNA-seq), different studies have elucidated the molecular architectures of different types of neurons and glial cells in human and mouse nervous system (Mayer et al., 2018; Li et al., 2020; Fan et al., 2020). With the most comprehensive and up-to-date molecular taxonomy of different cortical cell types, understanding of the underlying mechanisms/programming of neurogenesis and the cellular diversity in the mammal cortex become possible (Yuzwa et al., 2017; Nowakowski et al., 2017; Zhong et al., 2018; Bhattacherjee et al., 2019; Loo et al., 2019; Polioudakis et al., 2019; Fan et al., 2020). Increasing studies describe the transcriptional landscapes of early cortical development in human and mouse (Geschwind and Rakic, 2013; Silbereis et al., 2016; Zeisel et al., 2018; Bandler et al., 2022). However, current studies focus on validation of the findings/hypotheses generated from former mouse studies.

In this study, we investigated the temporal transcriptomic landscapes of neocortex at single-cell levels during multiple prenatal stages with published scRNA-seq datasets in mice. Our results revealed a neuronal transition directly from DLNs to ULNs (D-T-U) during cell migration from sub-ventricular zone (SVZ) to cortical plate (CP), specifically expressed genes in which showed different expression timepoints in human. It might potentially result in the differences of cellular compositions and transcriptional profiles in human and mouse adult PFC.

## Results

### Developmental trajectories of the projection neurons

We retrieved scRNA-seq datasets of an overall 144 samples from human and mouse neocortex sampled at multiple prenatal developmental stages [human: gestational weeks (GW) from 5.85 to 37 (*n* = 78); mouse: E11.5-P0 (*n* = 66), see Methods (Supplementary Tables S1–4)] for the analysis (reanalysis of UMAP shown in Supplementary Figure S1A). To minimize the potential batch-effects caused by different studies, we analyzed the datasets from one single study and replicated the analysis by using the other datasets independently as validation.

**FIGURE 1:**
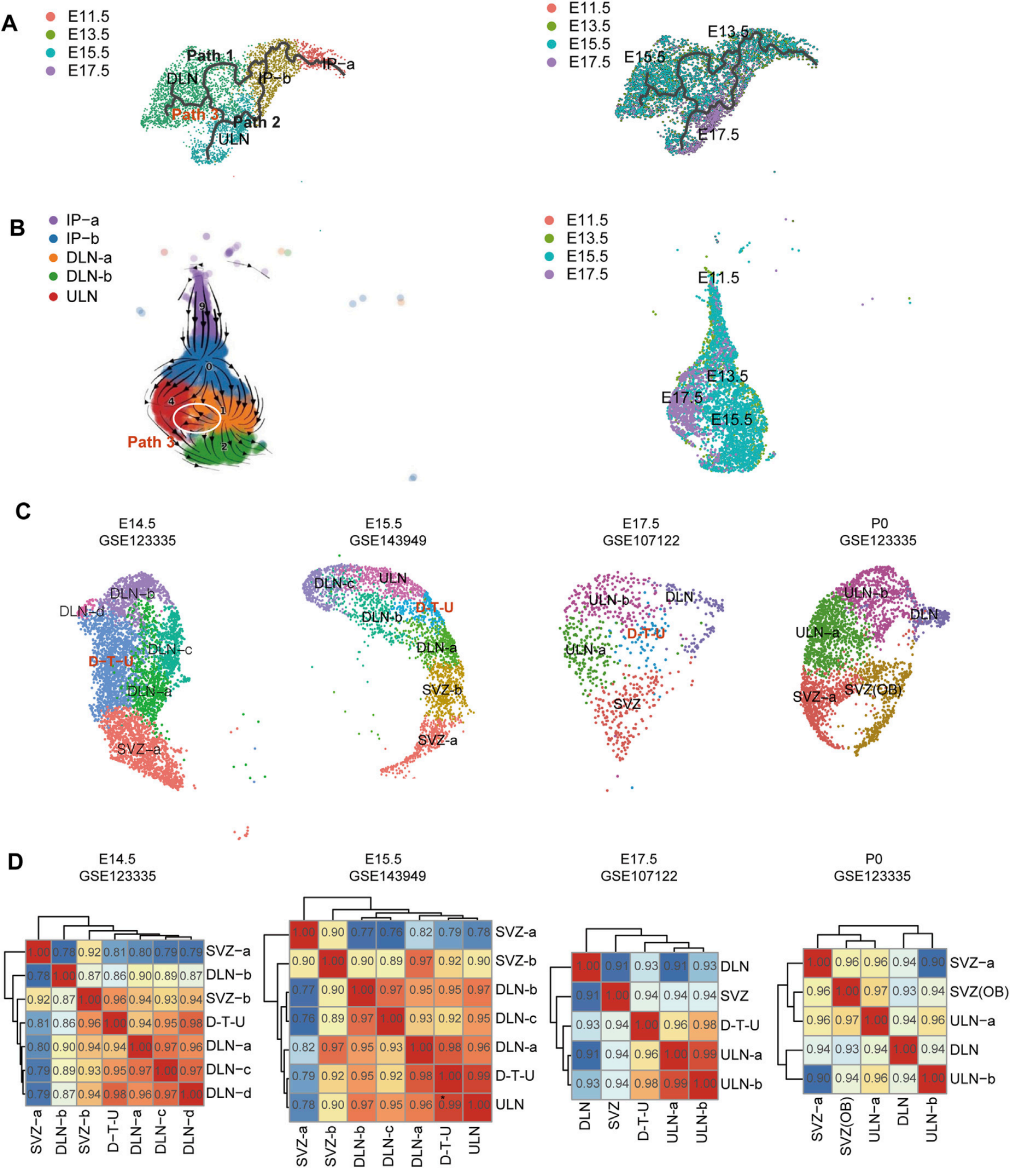
Developmental trajectories of projection neurons in mice. Pseudotime map generated from the mouse datasets with Monocle3 **(A)** and scVelo **(B)**. In left, the cells are labelled by the different cell types, while in right, cells were labelled by the sample collection timepoints. The additional path (path 3) was indicated by red fonts **(A)** and by a white circle **(B)**. **(C)** The transcriptional profiles of D-T-U subset in mouse excitatory neurons at E14.5, E15.5, E17.5 and P0. UMAP visualization of the excitatory neurons that were identified in mouse cortex cells. Cell cluster from D-T-U is indicated in red font in each figure. **(D)** Similarity matrix of expression among different cell clusters. Pairwise Pearson Correlations between clusters across principal components in mouse are provided. The Pearson Correlation Coefficient between each two cluster is shown accordingly.

We investigated the developmental trajectories of projection neurons in mouse neocortex by selecting the mouse datasets from Yuzwa’s study (Yuzwa et al., 2017) (Supplementary Figure S1A), which had the most comprehensive timepoints in prenatal stages (Supplementary Figure S1A). We selected those clusters with *NEUROD6* and *NEUROD2* (marker for excitatory neurons) (Tutukova et al., 2021; Bandler et al., 2022) identified (Supplementary Figure S2) and subjected them for the analysis of developmental trajectories by using Monocle3 (Qiu et al., 2017) and scVelo (Bergen et al., 2020), respectively. The results from Monocle3 indicated that there were three developmental branches, where progenitor cells differentiated into DLNs (Path 1) and ULNs (Path 2) (Figure 1A). Whilst an additional subpath of DLNs was identified showing an apparent tendency of cell transition from DLNs to ULNs (Path 3) (Figure 1A). The scVelo analysis also confirmed the same observation (Figure 1B). We further replicated the analysis in the datasets from Ruan’s study (Ruan et al., 2021) and yielded a similar finding (Supplementary Figure S1B), confirming such subpath likely occurred on/after E13.5 (Figure 1B). For human neocortical development, we selected human datasets from Fan’s study, include four lobes including frontal lobe (FL), parietal lobe (PL), occipital lobe (OL) and temporal lobe (TL) between GW10 and GW21, as these human ages are analogous to E11.5 to E17.5 mouse ages (Li et al., 2020). However, potentially biased developmental trajectories of projection neurons were observed (Supplementary Figure S1C), partially due to the biased cell composition presented in early gestational weeks (DLNs were dominant in GW10) and/or the insufficient cell number per sample (Nishimura et al., 2005; Yagishita et al., 2014). Therefore, the following analyses were focused on the data derived from mouse neocortex.

### Cell transition and migration of D-T-U

To enhance the differences of expressions between prenatal and postnatal stages in mouse cortex, we used the datasets from Loo’s study (Loo et al., 2019) with E14.5 and P0 available for the analysis. A subcluster of DLN (DLN-a in E14.5) in mice was with migratory transcriptional regulators (*Tima2*, *Pou3f2* and *Ptn*) expression detected (Supplementary Figure S3A) (Loo et al., 2019). Further unbiased clustering of these DLN-a cells revealed a subcluster (752 cells, accounting for 10% of mouse projection neurons). In this subcluster, expressions of both DLNs (*Bcl11b/Ctip2*) and ULNs markers (*Cux1* or *Satb2*) were simultaneously identified in a significant proportion of the cells (Supplementary Figure S3A). The presence of this subcluster might explain the third developmental subpath observed in the pseudotime analysis (we named as D-T-U cell cluster).

Further analysis was performed for the datasets from samples collected at each single timepoint (P0 from Loo’s study (Loo et al., 2019), E15.5 from Li’s study (Li et al., 2020), and E17.5 from Yuzwa’s study (Yuzwa et al., 2017)), all datasets from prenatal stages indicated the presence of DLN cell subcluster (D-T-U cell), which was absent in postnatal stage (P0, Figure 1C). In addition, we tested the similarity of expression patterns among different cell clusters. Pairwise Pearson Correlations between clusters in mouse were performed for the datasets within the same timepoint (Figure 1D). In E14.5, D-T-U cluster shared an expression pattern with different subclusters of DLNs and SVZ-b (progenitors of ULN), while in E15.5 and E17.5, the similarity of expressions between D-T-U with ULN cluster (indicated by the Pearson Correlation Coefficient) was higher than that between D-T-U with the other DLN subclusters. It indicated that the dynamic changes of transcriptional profiles of D-T-U subsets was accompanying the timing of developments/generations of neurons from different layers (*i.e.,* DLNs and ULNs).

**FIGURE 2:**
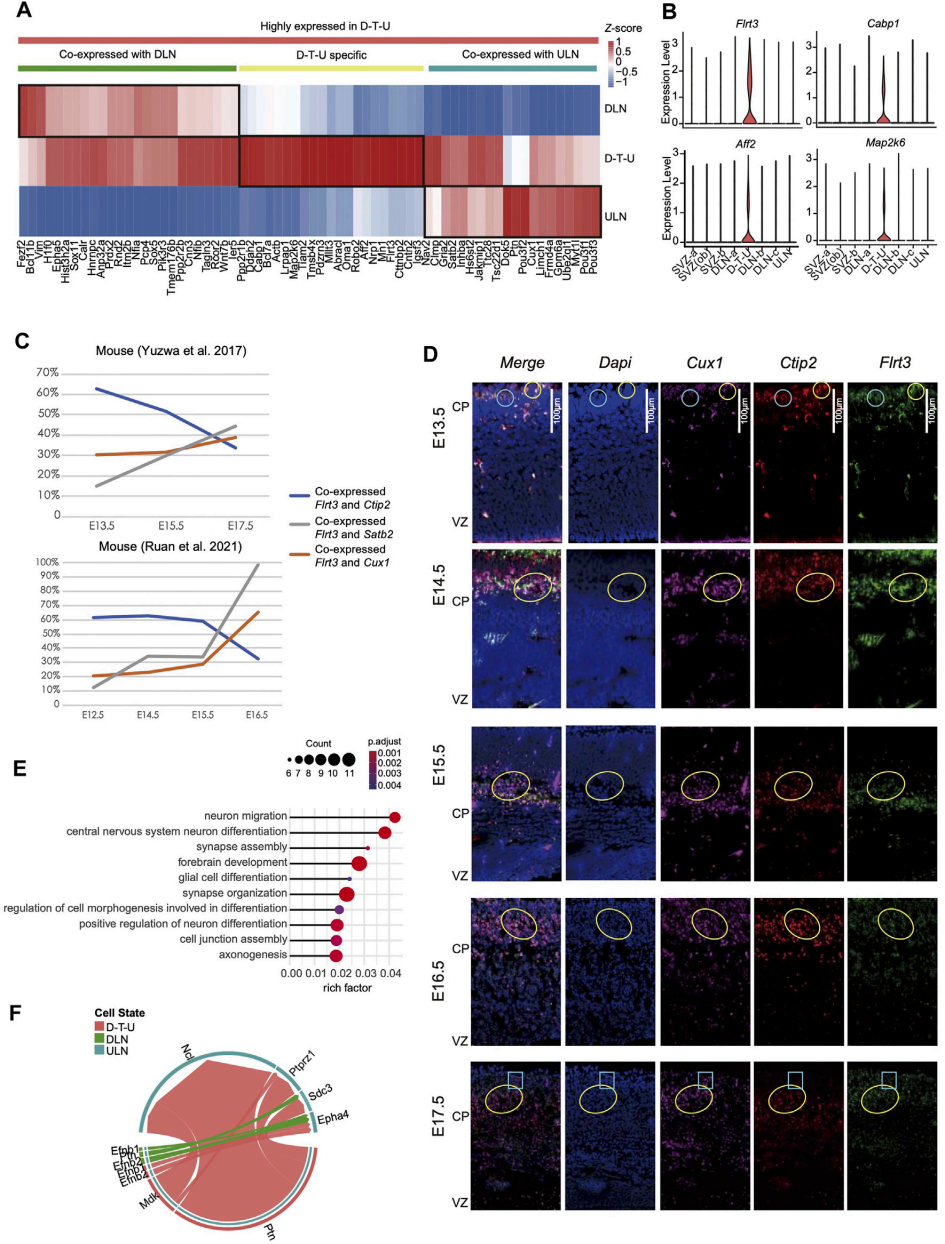
Differentially expressed genes identified in the D-T-U cell cluster in mice. **(A)** The relative expression levels (Z score) of 67 genes significantly highly expressed in D-T-U cluster among D-T-U, DLNs and ULNs cells. **(B)** Genes uniquely expressed in the D-T-U cell cluster. **(C)** Percentage of cells with co-expression of *Flrt3* and *Ctip2* (DLN marker), *Satb2* or *Cux1* (ULN maker) in two studies cross different timepoints (*X*-axis). **(D)** Co-immunostaining of ULN and DLN markers in mice. The staining results of E13.5, E14.5, E15.5, E16.5 and E17.5 mice are shown, respectively. The marker used for each figure is indicated in the upper panel and the cells with co-stained signal of *Cux1*, *Ctip2* and *Flrt3* are revealed by yellow circles in each figure, while the cells with co-stained signal of *Flrt3* with *Cux1* or *Ctip2* exclusively are indicated by blue circles/frames. **(E)** Enriched GO terms for upregulated genes in D-T-U compared to DLNs and ULNs in mouse. The *X*-axis shows rich factor, which refers to the ratio of number of genes with differentially expressed in this pathway dividing by the total number of genes in this pathway. **(F)** Comparison of the significantly enriched ligand-receptor pairs between D-T-U to ULNs and DLNs to ULNs. Different colors in the circle plot represent different cell groups and edge width in each circle represents the communication probability.

We further analyzed the specific expression patterns of the genes in cells from D-T-U cluster in E14.5 (Loo et al., 2019) with an attempt to identify the molecular mechanisms participating in this process. Sixty-seven differentially expressed genes (DEGs, Figure 2A) in D-T-U were identified by comparing the genes expressions in D-T-U with the ones in DLNs [DLN-a-a (exclusion of cells in D-T-U from DLN-a), DLN-b and DLN-c] and in ULNs, respectively. It included 22 DEGs significantly differentially expressed in D-T-U, 25 DEGs in ULNs, and 20 in DLNs. Among the 22 DEGs with significantly differential expression identified in D-T-U, *Flrt3*, *Cabp1*, *Aff2*, *Map2k6* and *Pdzrn3* were found to be specifically expressed in D-T-U (Figure 2B). The other 18 DEGs included *Ppp1r1b* (critical for dopamine-dependent striatal synaptic plasticity (Yagishita et al., 2014)), *Tiam2* (involved in the regulation of neuronal migration, neurite formation, growth-cone morphology, axon specification, and neuron polarity in mouse (Kawauchi et al., 2003; Nishimura et al., 2005)), *Pdzrn3* (required in radial glia for the regulation of lineage-autonomous and stage-specific gene expression programs that control the number and position of upper layer cortical projection neurons (Baizabal et al., 2018)), *Cntn2* (neuronal migration and axon fasciculation (Gurung et al., 2018)) and *Igsf3* (a novel regulator of neuronal morphogenesis (Usardi et al., 2017)). *Flrt3* has been reported to be involved in the regulation of cortical migration and sulcus formation in mouse (Seiradake et al., 2014; Del Toro et al., 2017), while induction of ectopic expression of *Pdzrn3* in E14.5 resulted in the observation of significantly higher number of *Cux1+* neurons in deep layer in P5 compared with negative control by cell migration analysis (Baizabal et al., 2018). It indicated that this D-T-U cell population might be involved in determination of the cortical neuronal position.

In order to investigate whether the transcriptional status of D-T-U subsets were altered during the migration process, we tested the rate of cells co-express layer-specific markers *Ctip2*, *Cux1* and *Satb2*, on the basis of expressing *Flrt3* (uniquely expressed in D-T-U) in datasets with multiple prenatal timepoints (Yuzwa et al., 2017; Ruan et al., 2021). The proportion of cells co-expressed *Ctip2* decreased during the time span, while the proportion of cells co-expressed *Cux1* and *Satb2* showed an opposite trend (Figure 2C). It potentially indicated a transition of DLN to ULN during the development.

To confirm the observations of neurons with co-expression of ULN and DLN markers as well as D-T-U specific markers in mice and the migration during development, we performed co-immunostaining of *Ctip2* (DLN specific marker), *Cux1* (ULN specific marker) and *Flrt3* for the mouse cortexes during the embryonic development (from E13.5 to E17.5). In E13.5-E15.5, cells stained with *Cux1* were predominantly located in SVZ and deep-layers, while cells stained with *Ctip2* were mainly located in deep-layers (Figure 2D). In E15.5-E16.5, cells stained with *Cux1* were presented in deep-layers and with a small proportion identified in upper-layers, while in E17.5, increasing number of cells stained with *Cux1* were observed in upper-layers. Whereas, during the development of cortex (E13.5 to E17.5), cells co-expressed *Flrt3*, *Ctip2* and *Cux1* also migrated together with ULNs (labelled by *Cux1*). It indicated that this cell population migrated from SVZ to the upper layer (*i.e.,* CP) during cortical development. Co-staining *Pdzrn3* with *Cux1* and *Ctip2* revealed a similar finding (Supplementary Figure S4) of the co-staining results of *Ctip2*, *Cux1* and *Flrt3*. Therefore, these results confirmed our finding from scRNA-seq analysis that the existence of subclasses showed DLNs and ULNs characteristics and expressed specific marker genes in mouse during fetal cortical development, namely D-T-U showing cell migration.

We further investigated the potential functions of other DEGs (Figure 2A). Among the 25 DEGs in DLNs (co-expressed with D-T-U), *Fezf2* and *Bcl11b* were the SCPN marker genes, and *Sox5* was CThPN marker gene. In comparison, among the 20 DEGs in ULNs (co-expressed with D-T-U), *Cux1* and *Satb2* were the well-established CPN marker genes, and *Inhba* was specifically expressed in upper-layer CPN (Molyneaux et al., 2009) (Supplementary Figure S3D). Particularly, those genes with high expression identified in deep-layer CPNs (such as *Fam19a2, Cdh13*, *Igsf21* and *Gnb4*) (Di Bella et al., 2021), the expressions of which were not detected in D-T-U (Supplementary Figures S5C, D). In addition, for those genes known to be specifically expressed in ULNs (particularly Layer 2 and 3; *Cux2*, *Lhx2* and *Pou3f2*) or DLNs (*Sox5*, *Bcl11b* and *Fezf2*), all of them shared similar expression patterns with the cells from D-T-U (Supplementary Figures S6, 7). Taking together, this result confirmed a specific cell transition distinguished from deep-layer CPNs.

GO enrichment analysis was performed with all 67 DEGs (Figure 2A), which indicated that DEGs were enriched in the processes of neuron migration and differentiation (Figure 2E). We further detected ligand-receptor combinations for the D-T-U cluster. Interestingly, cells in D-T-U cluster mainly expressed the migratory factors: Ptn and Mdk, compared to the other DLNs (Figure 2F). In addition, several receptor/ligand pairs (such as Ptn-Ncl, Ptn-Ptprz1, Ptn-Sdc3, Mdk-Ncl and Mdk-Ptprz1), associated with neuronal migration, were found to connect the D-T-U subsets with ULNs in mouse cortical projection neuron (Figure 2F).

### Investigation the similarities and differences of transcriptomic profiles of projection neurons in human and mouse neocortex

**FIGURE 3:**
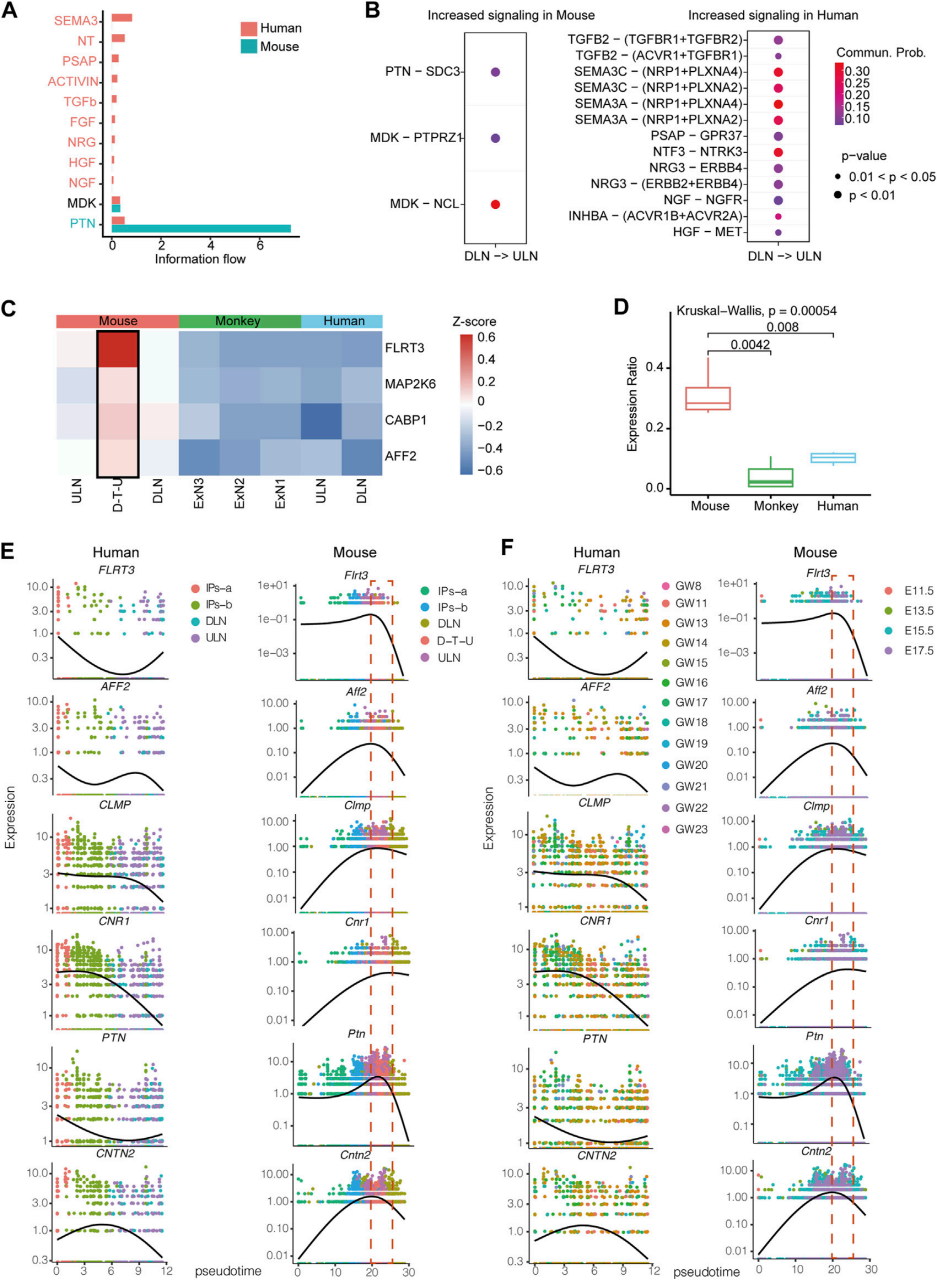
The differences of transcriptomic profiles between human and mouse prenatal stages. **(A)** Significantly enriched signaling pathways in humans and mice. The order is shown based on the differences of overall information flow within the inferred networks between human and mouse. The top signaling pathways colored in red are specifically enriched in humans, the middle one colored in black is equally enriched in human and mouse, and the bottom one colored in blue are specifically enriched in mouse. **(B)** All significantly enriched ligand-receptor pairs that contributed to the signaling sending from DLNs to ULNs. The dot color and size represent the calculated communication probability and *p*-values, respectively. *p*-values are computed based on one-sided permutation test. **(C)** Comparison of *Flrt3*, *Cabp1*, *Aff2* and *Map2k6* expression levels among mouse, human and monkey in the neocortex projection subgroups. **(D)** Comparison of the factions of cells with *Flrt3*, *Cabp1*, *Aff2* and *Map2k6* expressions identified between mouse D-T-U, human and monkey projection neurons subtypes. Whiskers in the boxplot represent minimum and maximum; unpaired Kruskal–Wallis test. Expression heatmap of highly dynamically expressed genes ordered across pseudo-time in human and mouse labelled by different cell populations **(E)** or different timepoints **(F)**.

To further illustrate cell communications among projection neurons in mice, we applied a CellChat algorithm to analyze the enrichment of interaction pairs (Jin et al., 2021). In addition, we utilized the available datasets from human cortex for the analysis, although the cell numbers in each sample were insufficient for the aforementioned trajectory analysis. The number of interaction pairs in mice was significantly increased in the information flow of PTN and MDK signaling pathways (Figures 3A, B). PTN was found to have significantly higher interaction-pairs in mouse compared with ones detected in humans. It has been known that PTN binding to *SDC3* gene to promote neurite outgrowth and migration (Sorrelle et al., 2017), while PTN is also involved in cell transformation, growth, survival, migration and angiogenesis (Sethi et al., 2015). In addition, MDK, which binds to *PTPRZ1* known to promote neuronal migration and embryonic neurons survival (Maeda et al., 1999), and binds to *NCL* to promote nuclear localization, endothelial cell migration, and cell survival (Koutsioumpa et al., 2013), were found with comparable number of interaction-pairs between human and mouse (Figure 3B). Furthermore, we identified a significant increase of interaction-pairs between DLNs and ULNs in human (Figures 3A, B), including semaphorins (SEMA3C and SEMA3A, implicated in axon repulsion, dendritic branching and synapse formation of central neurons via binding with its receptor NRP1 (Shelly et al., 2011)), NT (NTF3, controlling survival and differentiation of mammalian neurons (Usui et al., 2012)), PSAP (a neurotrophic factor (Li et al., 2017)), and TGFb (TGFB2, regulating neuron cell apoptosis or death (Hashimoto et al., 2005; Meyers and Kessler, 2017)).

To determine whether these cells exhibited a rodent-specific manifestation, we selected the datasets from human (Nowakowski study) (Nowakowski et al., 2017) and 110 post-conception days macaque neocortex (Zhu et al., 2018) for comparison. Four marker genes *FLRT3*, *AFF2*, *MAP2K6* and *CABP1* with high expression in mouse D-T-U subsets were found to have low expression levels and low cell percentage with expression identified (referred to the percentage of cells with expression of that gene identified) in human and monkey (Figures 3C, D).

Further, we performed co-regulation analysis, and identified 206 genes co-expressed with *Flrt3* during development. By pseudotime analysis and comparing the expression patterns of these genes, we identified six genes (*Flrt3*, *Aff2, Cntn2*, *Clmp*, *Cnr1* and *Ptn*), which showed a potential opposite temporal trend between humans and mice. In humans, they were likely expressed in early cortical development (highly expressed in progenitor cells, GW11-GW17), while in mouse, they were expressed in the later stages (highly expressed in D-T-U, E15.5 and E17.5) (Figures 3E, F). *Flrt3* (Del Toro et al., 2017), *Ptn* (Sethi et al., 2015) and *Cntn2* (Gurung et al., 2018) were known to regulate neuronal migration and axon fasciculation, while *Clmp* and *Cnr1* were related to cognitive disruption and abnormal behavior in humans and mice (Evans et al., 2016; Jang et al., 2020). In particular, as *Cnr1* is known to show an age-related cognitive impairment (Bilkei-Gorzo et al., 2005), the presence of D-T-U in a time-sensitive timepoint (E14.5-E17.5) might be a supplement to regulate the cognitive functions in later stage in mice, a different mechanism of cortical development in human. We further performed co-expression analyses of the six mice D-T-U specific genes (*Flrt3*, *Aff2*, *Clmp*, *Cnr1*, *Ptn* and *Cntn2*) from E14.5 to E17.5 mouse cortexes. The results showed that all these six genes were co-expressed in the D-T-U cell population in datasets from each timepoint (Supplementary Figure S8), indicating a potential group of genes important for mouse fetal cortical development.

### Differences of expressions in adult projection neurons between human and mouse

Through the forementioned analysis, we identified a small cell population showing cell direct transition from DLNs to ULNs in mice and the genes specifically expressed in this cell cluster showed potentially different expression timepoints between humans and mice (Figure 3). We further investigated whether such differences correlated with the differences in adult PFC between humans and mice. We used SCTransform for normalization and Seurat version 4 for batch correction (see *Methods*).

**FIGURE 4:**
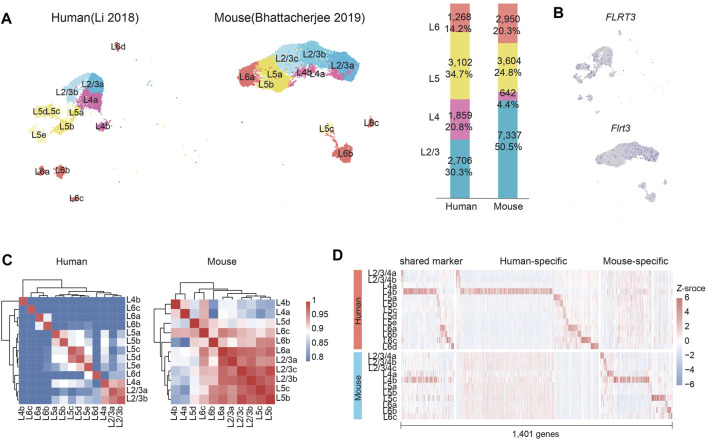
The differences of transcriptional profiles of projection neurons in human and mouse adult PFC. **(A)** Clustering result of projection neurons in independent PFC datasets of human (left) and mouse (right). Bar plot shows the percentage of cells in each layer in human and mouse: L2/3 in blue, L4 in pink, L5 in yellow and L6 in red. **(B)** UMAP visualizations of the expression of *FLRT3* in adult PFC projection neurons in human (upper) and mouse (lower). **(C)** Pearson intra-species correlations of transcriptomic profiles of projection neuron clusters in human (right) and mouse (left). **(D)** Shared or species-specific differentially expressed genes for all projection subclasses.

We compared the cell types and expressions in projection neurons in human adult PFC (*n* = 3) (Li et al., 2018) with the ones in mouse (*n* = 12) (Bhattacherjee et al., 2019) by applying unbiased clustering based on uniform manifold approximation and projection (UMAP; see *Methods*) and spectral K nearest-neighbor graph-based clustering (Butler et al., 2018). We identified 13 clusters of cells in human (two clusters of Layer 2/3; two clusters of Layer 4; five clusters of Layer 5; and four clusters of Layer 6) and 11 clusters of cells in mouse (three clusters of Layer 2/3; two clusters of Layer 4; three clusters of Layer 5; and three clusters of Layer 6). The finding of Layer 4 in mouse adult PFC against the finding from a previous mouse study, in which L4 was not detected (Bhattacherjee et al., 2019) (Figure 4A). Two subtypes of mouse Layer 4 were identified, and both showed a high expression of gene *Rorb* (marker of Layer 4 stellate neurons).

For those specific markers identified in mouse prenatal D-T-U subclusters, they were expressed in different layers in human and mouse. For instance, *Flrt3* gene was found to expressed in mouse Layer 2, while lower expression of *FLRT3* was observed in human Layer 5 (Figure 4B). In addition, *PDZRN3* was found to be mainly expressed in DLNs in humans but in ULNs in mice (Supplementary Figure S9). However, most gene were with dynamic expressions cross different timepoints, our findings indicated that the presence of D-T-U in mouse cortical development might result in the differences of cellular compositions and transcriptional profiles (such as different layers for gene expression) in human and mouse adult PFC.

We further investigated the transcriptional profiles of adult projection neurons in humans and mice. As the correlation of gene expression would reflect histological distance during development, we compared the transcriptomic profiles among all cells from different layers in human and mouse. Interestingly, in mouse adult PFC, the transcriptomic profiles of all cell types from different layers (among Layer 2/3, 5 and 6) showed high correlation (Figure 4C), while in human, the transcriptional profiles showed a remarkable laminar organization between different layers (Figure 4B).

In addition, the projection neurons subclasses expressed 5 to 800 marker genes, and there was only a small proportion (*n* = 169, 12.1%) sharing the same expression patterns between human and mouse (999 and 233 were specific to human and mouse, respectively). Such finding was consistent with the reported primary motor cortex (MOp) study result (BRAIN Initiative Cell Census Network BICCN, 2021). In particular, Layer 4b had a core set of 91 markers that were conserved between human and mouse (Figure 4D), suggesting a high degree of evolutionary consistency in the subgroup Layer 4b between human and mouse.

Taken together, we identified a cryptic cell population showing neuron transition and migration (D-T-U) during mouse fetal cortical development, which potentially resulting in the differences of cell types and genes expressions identified in adult PFC between humans and mice.

## Discussion

**FIGURE 5:**
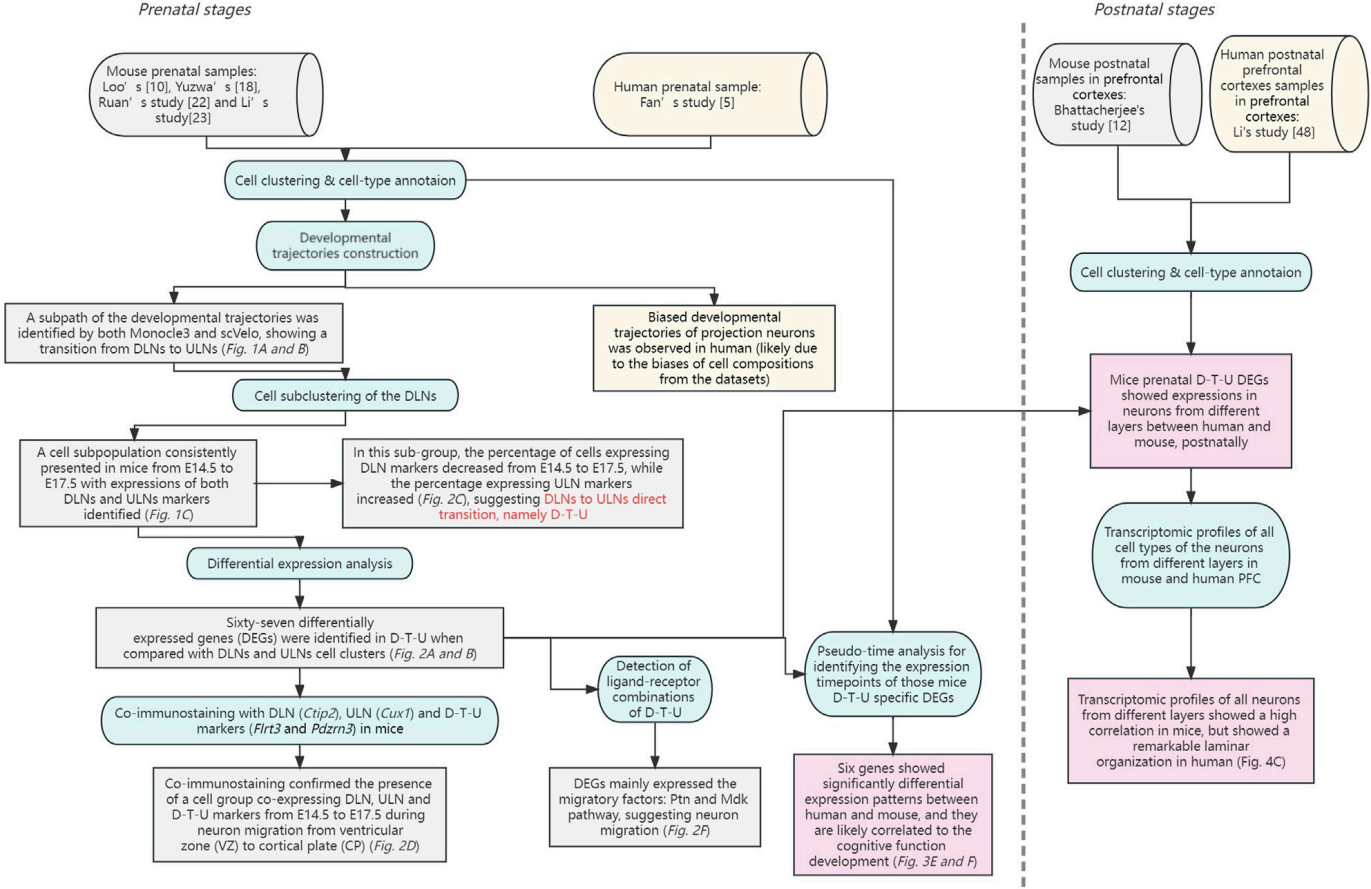
A brief summary of the analytical and experimental workflow with key findings provided for this study. Two phases of this study are shown separately by a dotted line. The datasets used for the analyses (with references provided) are shown in cylinders. In addition, the analytical or experimental steps are shown in circular rectangles, while the key findings (with corresponding subfigures) are shown in rectangles. The datasets and findings in mouse alone are shown in grey, while the datasets and findings in human alone are shown in yellow. For the comparison results, they are shown in pink.

In this study, we systematically characterized the temporal transcriptional landscapes of different cell types in corticogenesis in mice by single-cell analysis and identified a D-T-U cell population showing direct DLNs to ULNs transition and migration (Figure 5).

It has been proposed that the birthdate of a neuron is highly in accordance with its final neocortical laminar location, and the same type of laminar neurons share common projection targets. Current knowledge of developing DLNs and ULNs is that early-born neurons likely differentiated into DLNs, while later-born neurons migrated outward the deep-layer to establish ULNs (Greig et al., 2013; Ohtaka-Maruyama and Okado, 2015; Ohtaka-Maruyama and Okado, 2016). It has been known that induction of *Foxg1* by *FGF8* represses *Tbr1* in the layer transcriptional network, switching the progenitor fate to DL production. Later on, the transition of production from DL to UL neurons is regulated by signals propagated from postmitotic DLNs, terminating DLN production through negative feedback (Toma and Hanashima, 2015). In other words, ULNs were generated from progenitor cells upon the completion of DLNs generation. We reconstructed the molecular developmental trajectories of projection neurons in mice. In mouse cortex, an additional subpath of DLNs was identified showing an apparent tendency of cell transition from DLNs to ULNs (Figure 1). It indicated a proportion of cells in mouse cortical development might follow a “nested multipotential lineage” model (Greig et al., 2013).

Our analysis indicated that a small cell population showing cell transition from deep-to upper-layer neurons (namely D-T-U) was first detected on/after E13.5 and was absent after E17.5. They were transient cells exhibiting both DLN and ULN characteristics and cell-type-specific function genes, which has been confirmed by co-immunostaining. Interestingly, our study revealed that the earliest time of observing Layer 2/3 (L2/3) ULNs was as early as in E14.5, which might be earlier than the current knowledge (such as E15.5) (Hagey et al., 2020; Heavner et al., 2020; Di Bella et al., 2021). In addition, the interaction pairs were found to be significantly enriched in mouse in the PTN and MDK signaling pathways, both of which are known to promote neuronal migration (Jung et al., 2004; Elahouel et al., 2015; Tang et al., 2019). Particularly, the PTN pathway is involved in cell transition (such as epithelial-mesenchymal transition) (Perez-Pinera et al., 2007). All evidence together supported the neuronal migration and transition from DLNs and ULNs specifically in mouse cortical development.

To further investigated the impact of the presence of D-T-U cell migration and transition in mouse compared with human, we firstly investigated the expression timepoints of those genes with co-expression with *Flrt3* (D-T-U cluster specifically expressed gene). The results indicated that genes related to cognitive development were expressed in different developmental stages in humans and mice (including *Cnr1* an age-related cognitive impairment gene). In addition, D-T-U specific markers genes were found to have different expression patterns in projection neurons in human and mouse adult PFC. This indicates human and mouse corticogenesis might follow different mechanisms.

Limitations remain: (1) The results were generated by using published datasets, in which batch effect or sampling bias might exist. To minimize the potential batch-effects caused by different studies, we performed our analysis with the datasets from one single study and replicated with another independently as validation. The finding of three developmental sub-paths in mice was consistently observed from the pseudotime analysis by two software Monocle3 and scVelo, independently, among multiple datasets. Whilst the cell cluster co-expressing DLN and ULN markers was also consistently observed from the datasets collected in the same timepoint from multiple datasets. Such findings were further confirmed by co-immunostaining experiments. It indicates that the batch effect was minimal. In addition, as some of the datasets in prenatal stages used in our analysis did not specify the neocortical region, it prevents our further understanding on the differences in the development of DLNs and ULNs in different cortical regions. Further study to collect neurons from different cortical regions for comparison is warranted. (2) The results from pseudotime analysis might be affected by the sample collection timepoints. However, our result did not represent the convergent patterns of gene expression that occur in parallel in different cell populations. By using the datasets from samples collected at each single timepoint from multiple studies, we consistently identified a DLN cell subcluster (D-T-U cells) in prenatal stages, which was absent in postnatal stage. The presence of this subcluster might explain the third developmental subpath observed in pseudotime analysis. In addition, the results from different datasets consistently showed a decreasing of cells co-expressing *Flrt3* and *Ctip2* (DLNs) but increasing of cells co-expressing *Flrt3* and *Cux1* (ULNs) from E14.5 towards E17.5, which were confirmed by co-immunostaining. Furthermore, we tested the similarity of expression patterns among different cell clusters. Pairwise Pearson Correlations between clusters in mouse indicated that D-T-U cluster shared an expression pattern with different subclusters of DLNs and SVZ-b in E14.5, while in E15.5 and E17.5, the similarity of expressions between D-T-U with ULN cluster (indicated by the Pearson Correlation Coefficient) was higher than that between D-T-U with the other DLN subclusters. It indicated that the dynamic changes of transcriptional profiles of D-T-U subsets was accompanying the timing of developments/generations of neurons from different layers (*i.e.,* DLNs and ULNs). Therefore, both analytical and experimental results support our finding of this cell transition and migration. (3) Analysis of the developmental trajectories and the experimental validation were not conducted in human samples. Although pseudotime analysis indicated that there were six genes (mouse D-T-U specific markers) showed a potential opposite temporal trend between humans and mice during fetal development, we cannot exclude the possibility of existing D-T-U cell population in humans but with different specific expression markers. Due to the insufficient cell numbers in each sample from publicly available datasets from human cortex for the trajectory analysis as well as the difficulty of collecting human fetuses for validation, we confined our analyses to mouse data instead. Nonetheless, further study with human fetal cortical samples is warranted.

In conclusion, our single-cell analysis characterized the temporal transcriptional underpinnings of corticogenesis in mouse neocortex and identified a specific cell population (namely D-T-U) showing direct DLNs to ULNs transition and migration. It indicates that the underlying mechanism of corticogenesis might be different between humans and mice, and likely resulting in the differences of cell compositions and transcriptional profiles (in different layers) in adult PFC of human and mouse.

## Methods

### Ethics approval

All animal studies were approved by the University Animal Experimentation Ethics Committee (AEEC, 21-299-MIS), The Chinese University of Hong Kong.

### Dataset usage in this study

We collected the published datasets of single-cell RNA sequencing related to human and mouse prenatal and postnatal cortical development with a total number of 159 samples (human: *n* = 81 and mouse: 78, Supplementary Table S1). It includes samples collected in multiple prenatal timepoints [GW5.85-GW37 in human (*n* = 78), and E11.5-P0 in mouse (*n* = 66)], and in adult PFC (human: *n* = 3 and mouse: *n* = 12, Supplementary Tables S2, S3) (Yuzwa et al., 2017; Nowakowski et al., 2017; Li et al., 2018; Bhattacherjee et al., 2019; Loo et al., 2019; Polioudakis et al., 2019; Li et al., 2020; Fan et al., 2020).

### Clustering and cell-type identification

For each individual sample, Seurat v4.0.5 (https://satijalab.org/seurat/(Stuart et al., 2019)) was used to identify the expression matrices following by two-round clustering. Firstly, to eliminate biases generated due to poor data quality, we excluded (1) those genes with expressions identified in less than 5 cells; or (2) those cells with less than 500 genes detected. Data was then normalized to a total of 1 × 10^4^ molecules per cell based on the sequencing read-depth, and to control the strong relationship between variability and average expression, variable genes (defined as outliers) were identified by the “FindVariableGenes” function with the parameters set as “selection.method = “vst”, nfeatures = 2000.” In addition, the potential batch effect from this sample was mitigated by using the ScaleData function of Seurat. Principal component analysis (PCA) was further used to identify the top 30 PCs (Seurat ‘RunPCA’ function), which were further subjected for constructing a SNN network (spiking neural network). A graph-based clustering approach, louvain algorithm, was applied to identify cell clusters with marker genes. UMAP was used to visualize the clustering results.

In addition, to identify clusters of cells by a SNN modularity optimization-based clustering algorithm, we set the resolution parameter on the FindClusters function (Seurat) at 0.8. To avoid overfragmented clustering, we merged clusters with less than 10 DEGs based on the cutoff value “*p*_value <0.01, avg_LogFC >1” into one cluster. In this way, there were at least 10 DEGs between any two clusters. Subsequently, we identified five major cell types: progenitor, projection neurons (DLNs: L5/6 and ULNs: L2/3/4), interneuron and astrocytes, according to the classical markers and the previously published meta file (Nowakowski et al., 2017; Li et al., 2018; Bhattacherjee et al., 2019; Loo et al., 2019; Polioudakis et al., 2019; Fan et al., 2020) (Supplementary Table S4) if the marker gene was detected in over 80% of the cells in a cluster. As described in our previous study (Zhou et al., 2023). For further analysis with D-T-U cluster, the same analysis was applied for the cells from the cell cluster derived as DLNs to identify subclusters with FindClusters.

### Differentially expressed genes (DEGs) analysis

For gene differential expression analysis, we first set a unit as 1 cell type in a particular sample. For the expression level of a gene in each unit, we set it as the mean value of the expression levels of this gene identified among all cells from this cell type in this sample. We then defined a DEG as a gene with significantly differential expression identified in a unit (1 cell type in a particular sample) when comparing with the expression levels from all the other units, using the “FindMarkers” and “FindAllMarkers” module (min.pct = 0, logfc. threshold = 0, test. use = “wilcox”) from Seurat R package. Significantly upregulated or downregulated genes were identified based on the threshold of FDR (false-positive-rate) < 0.01 and FC (fold-change) > 1.5. For identifying genes that were spatially or temporally regulated, we used false-discovery-rate Q value of <0.01; log_2_-fold-change >1 and min. pct >0.25 instead. A gene list of DEGs was then generated by collecting all DEGs among the selected cases (from different timepoints or different studies). Further clustering of gene expression was conducted based on this gene list.

### Gene Ontology enrichment analysis

We used the clusterProfiler (Wu et al., 2021) R package to conduct Gene Ontology (GO) enrichment analysis to identify any biological processes or molecular functions enriched from the gene-set. For those with more than one gene-set subjected for the analysis, enrichGO and compareCluster were used. Simplify function was then used to remove redundant GO terms (cutoff = 0.7).

### Construction of single-cell trajectories for the projection neurons

The R package Monocle 3 was applied to construct single-cell pseudotime trajectories in human and mouse projection neurons (Darmanis et al., 2015). The “as.cell_data_set ()” function in SeuratWrappers R package was used to convert the Seurat object to a CellDataSet object, and the “learn_graph” module was used to simulate the developmental paths. The “plot_cells” function was then applied to visualize the developmental lineage(s). The python package scVelo was used to validate our result by estimate RNA velocity (Bergen et al., 2020). The “scv.tl.velocity” function was used to determine the unspliced/spliced phase trajectory for each gene, and “scv.tl.velocity_graph” function was used to construct the developmental path(s). Subsequently, the “scv.pl.velocity_embedding_stream” function was applied to visualize the developmental path(s).

We used the data matrix from Seurat with 2,000 highly variable genes as input for the pseudo-time order and identified genes with differential expression among distinct cell types with “differentialGeneTest”. “DDRTree” was applied to reduce the dimensional space and “orderCells” was used to sort the cells. To investigate genes correlated with a target gene (e.g., *Flrt3*), we utilized the “find_gene_modules” function. In addition, “plot_genes_in_pseudotime” was used to show gene expression dynamics during pseudotime.

### Mouse handling

Pregnant female C57BL/6J mice were supplied by the Laboratory Animal Services Centre (LASEC), the Chinese University of Hong Kong. Mice were maintained under a 12 h:12 h light/dark cycle at 21 ± 1°C and given standard chow and water *ad libitum*. All animal procedures were approved by the University Animal Experimentation Ethics Committee (AEEC), The Chinese University of Hong Kong.

### Validation by co-immunofluorescence staining

Pregnant female mice with embryonic gestational day from E13.5 to E17.5 were anaesthetized and perfused with Dulbecco’s phosphate buffered saline (PBS) and then 4% paraformaldehyde (PFA) in PBS. Mouse embryonic brains were dissected with ice-cold PBS and fixed with 4% PFA in PBS overnight followed by dehydration with 15%, and 30% sucrose overnight respectively at 4°C. After TissueTek^®^O.C.T.™ compound filtration overnight, the TissueTek^®^O.C.T.™-embedded mouse embryo brains at −20°C were sectioned into 10 µm using Epredia Cryostar NX70 Cryostat with Height Adjustment.

The sagittal embryonic brain sections were pretreated with methanol for 10 min and subsequently permeabilized and blocked with 0.2% Triton X-100/PBS and blocking buffer (10% normal goat serum (Thermo Fisher, 50062Z) and 0.2% Triton X-100) at room temperature for 40 min, respectively. After briefly washed with PBS, brain sections were incubated with primary antibodies, including mouse anti-*Cutl1*/*Cux1*/*Cdp* Antibody 1/25 (Santa cruz, 514008), rat anti-*Ctip2* antibody 1/200 (Abcam, 18465), rabbit anti-*Flrt3* antibody 1/150 (Invitrogen, PA5113445), and rabbit anti-Pdzrn3 antibody 1/100 (Invitrogen, PA5117726), diluted in 10% normal goat serum at 4°C overnight. After three washes with PBST (PBS contains 0.2% Tween-20), the brain sections were incubated with Alexa Fluor-conjugated secondary antibodies all diluted 1:500 in 10% normal goat serum at room temperature for 2 h. Secondary antibodies used were goat anti-rabbit IgG (H + L) antibody, Alexa Fluor™ 488 (cell signaling, 4412s), goat anti-rat IgG (H + L) antibody, Alexa Fluor™ 555 (Thermo Fisher, A-21434), donkey anti-mouse IgG (H + L) antibody, Alexa Fluor™ 647 (Thermo Fisher, A-31571). After three washes with PBST, the brain sections were mounted with *Dapi* to stain nuclei at room temperature for 5 min. Immunofluorescence was visualized with the Olympus Fluoview FV1200 SIM Confocal Microscope with FV10-ASW program.

### Analysis of the inter-lineage interactions

We used CellChat to perform systematic analysis of inter-lineage interactions within the neocortex (Jin et al., 2021). Through manifold learning and quantitative contrasts, CellChat classified the signaling pathways, and delineated the shared and species-specific pathways between humans and mice. We only selected those pathways where ligands and receptors communication probabilities were greater than 10% and *p*-value less than 0.05. Visualization of the ligand-receptor interactions were performed with “rankNet”, “netVisual_bubble” and “netVisual_chord_gene” functions from CellChat.

### Single-cell regulatory network inference and clustering using SCENIC

We performed SCENIC with the raw counts following the proposed workflow with the default parameters (Aibar et al., 2017). We used the AUCell value (score of the activity of each regulon in each cell) to identify target(s) of a regulon: the higher the AUCell value was identified if targets of a regulon matched the highly expressed genes in a certain cell was better.

### Projection neuron correlations in adult PFC

Expression matrices from samples in adult PFC were calculated and normalized by using the AverageExpression module from Seurat package (Stuart et al., 2019). Firstly, we applied the FindVariableFeatures function to identify 2,000 highly variable expressed genes in human and mouse, respectively. We then calculated the relationship between these 2,000 highly variable expressed genes and the others from the raw matrix (Stuart et al., 2019). The Pheatmap was further performed for the clustering. Pearson correlations were performed by comparing projection neuron clusters between humans and mice.

### Statistical analysis

Statistical analysis was performed using R (version 3.6.1). The Wilcoxon rank-sum test, Kruskal−Wallis test and Student’s t-test were used. Sample size and *p*-values were also provided.

## Data Availability

The original contributions presented in the study are included in the article/Supplementary Material, further inquiries can be directed to the corresponding authors.
