# Supplementary material for: Single-cell analysis reveals specific neuronal transition during mouse corticogenesis
Source: Front Cell Dev Biol. 2023 Nov 6;11:1209320. doi: 10.3389/fcell.2023.1209320 (PMC10657809; doi:10.3389/fcell.2023.1209320)
Supplement: Supplementary file 1 [file DataSheet2.PDF]

Supplementary Materials

Supplementary Figures

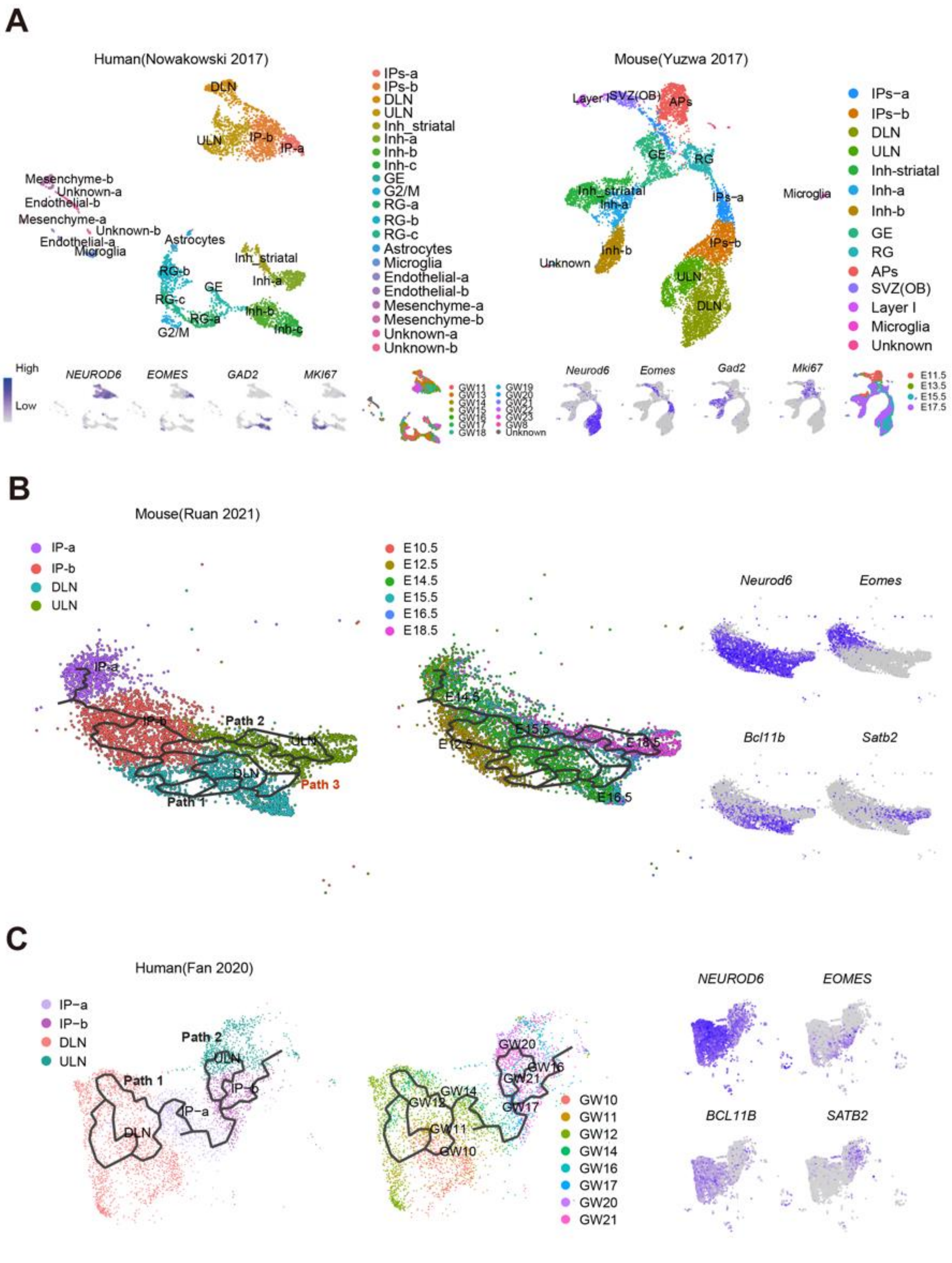

Supplemental Figure S1. Construction of developing lineage of excitatory neurons. (A) UMAP visualization of the clusters identified in cells from fetal cortex in human (left) and mouse (right). Each dot in the overall PCA is shown with the corresponding cell type (with marker genes expressed detected). Cells with expressions of four canonical marker genes (*NEUROD6*, *GAD2*, *EOMES*, and *MKI67*) are presented in the bottom by showing the cells with expression of each gene identified (UMAP projection). (B) Pseudotime map generated from mouse datasets from Ruan's study (Ruan 2021), and (C) from a human dataset from Fan's study (Fan 2020). In left, the cells are labelled by the different cell types [the progenitor cells from ventricular zone (VZ) and subventricular zone (SVZ), and excitatory neurons (DLNs and ULNs), Path3 is indicated in red font, while in the middle, the cells are labelled by the sampling timepoints. Cells with expressions of four canonical marker genes detected are shown on the right.

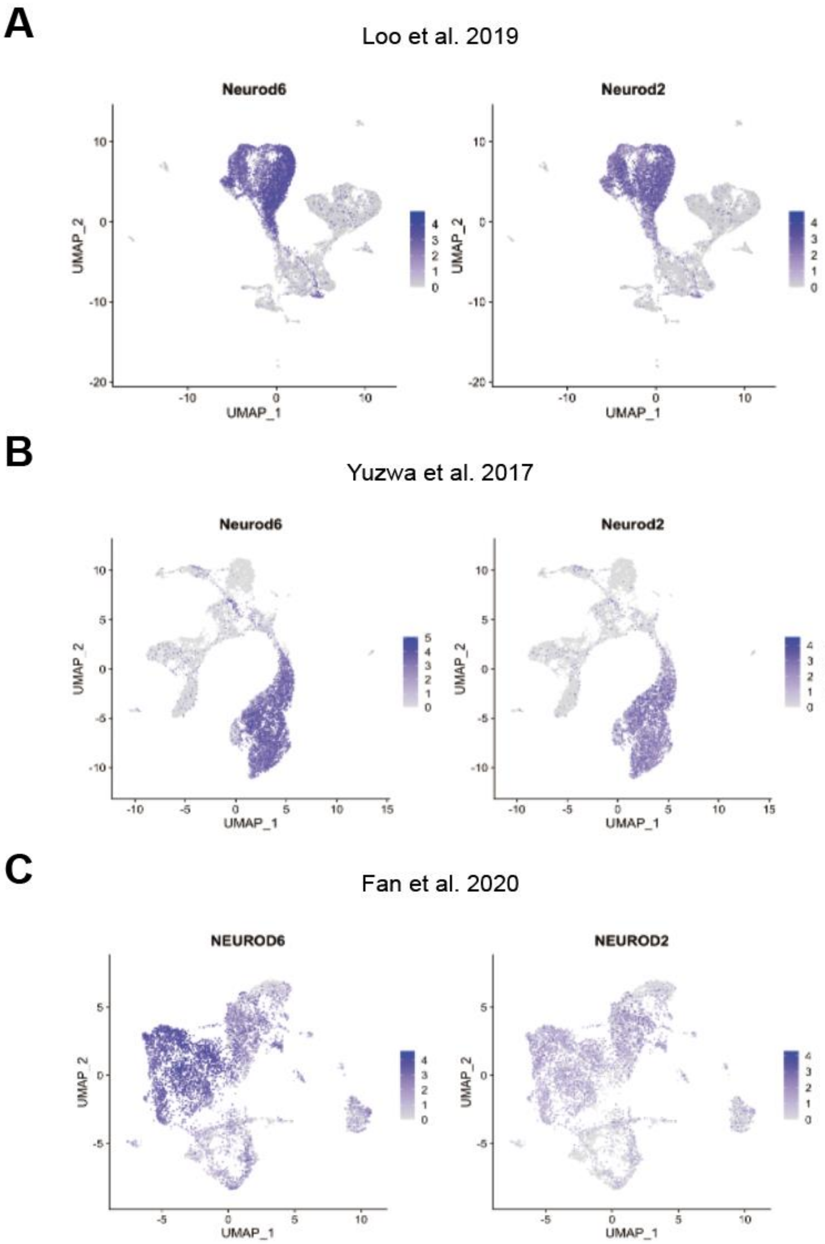

Supplemental Figure S2. Comparison of NeuroD family expression from human and mouse fetal neocortex datasets. Cells with expression of *Neurod6* (left) and *Neurod2* genes (right) identified are labelled in Loo's (A), Yuzwa's (B) and Fan's studies (C). All studies show a consistent genes expression pattern between two genes.

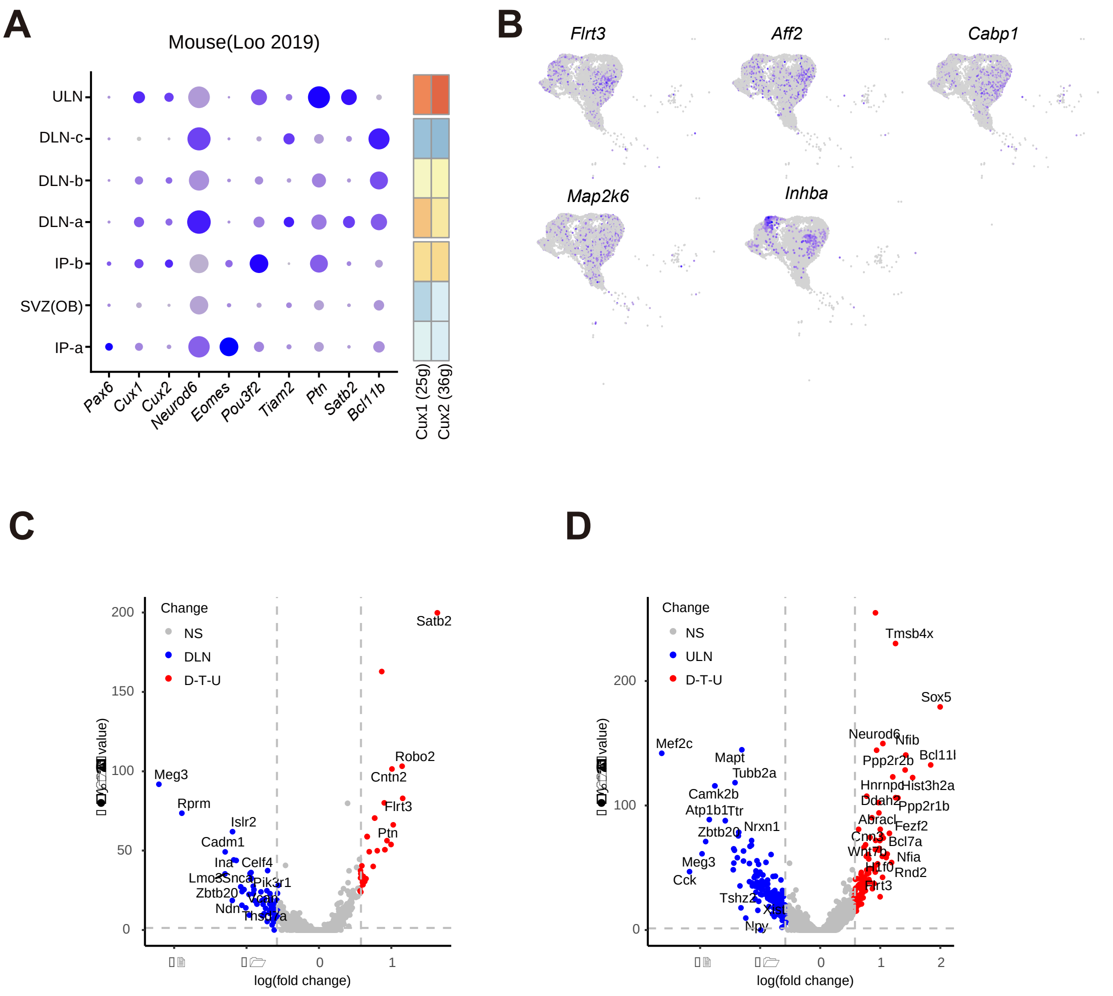

Supplementary Figure S3. Molecular characteristics of the D-T-U cluster. (A) Expression of eight known marker genes in mouse. In each subfigure, Dotplot shows the expression levels of each marker in each cell cluster: percentage of cells with expression of each marker is shown as the scale of circle; and average expression level is shown ranging from light to dark blue. In the right side of each subfigure, heatmap shows the AUCell scores (the activity of each regulon in each cell) of transcription factor: *Cux1* and *Cux2*. (B) UMAP projection of *Flrt3*, *Cabp1*, *Aff2*, *Map2k6* and *Inhba* expression in mouse excitatory neurons. (C) Comparison of differentially expressed genes between D-T-U versus all DLNs in mouse, and (D) between D-T-U and ULNs. NS refers to no significance identified.

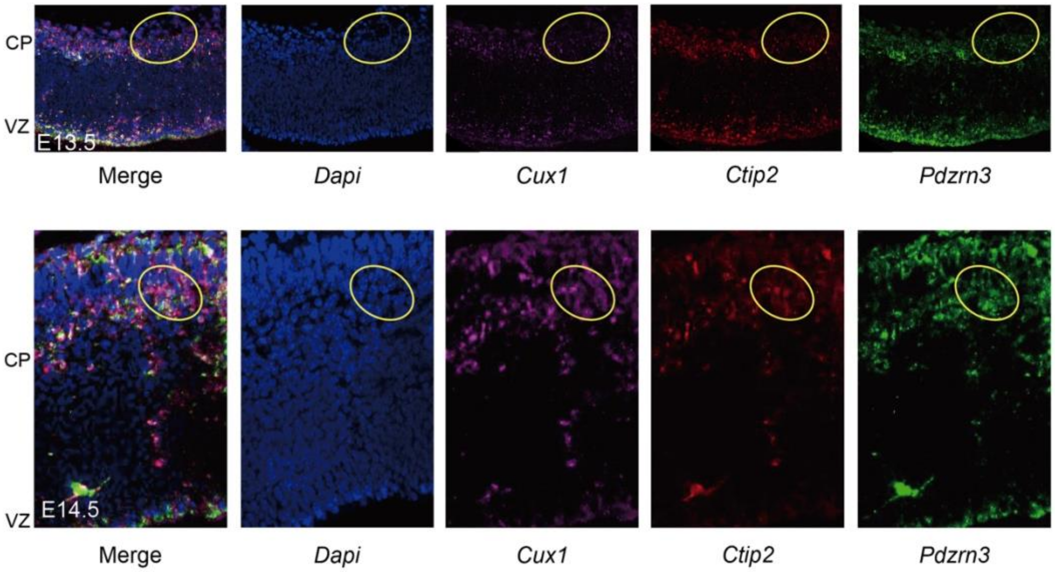

Supplementary Figure S4. Co-immunofluorescence of the D-T-U cluster. Co-immunostaining of ULN and DLN markers in mice. The staining results in a E13.5 and E14.5 mouse with different markers are shown, respectively. The marker used for each figure is indicated, and the regions with co-stained signal of *Cux1*, *Ctip2* and *Pdzrn3* are revealed by circles in each figure.

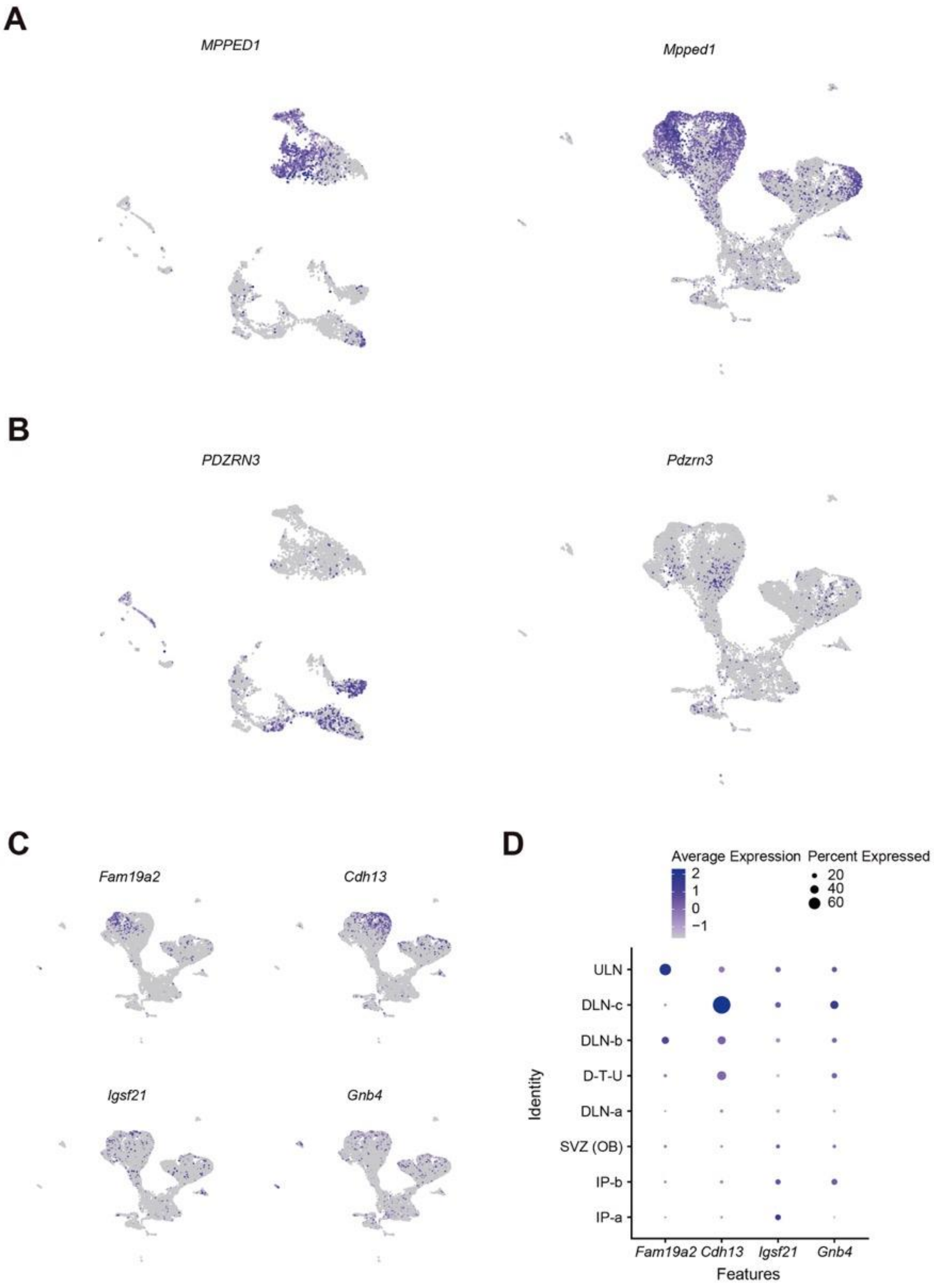
A
MPPED1
Mpped1
B
PDZRN3
Pdzrn3
C
Fam19a2
Cdh13
Igsf21
Gnb4
D
Average Expression
2
1
0
−1
Percent Expressed
20
40
60
ULN
DLN-c
DLN-b
D-T-U
DLN-a
SVZ (OB)
IP-b
IP-a
Identity
Fam19a2
Cdh13
Igsf21
Gnb4
Features

Supplementary Figure S5. Cell distribution with *Mpped1* or *Pdzrn3* expression identified. Cells with (A) *Mpped1* and (B) expression are shown in a UMAP visualization of the clusters identified in cells from fetal cortex in human (left) and mouse (right). (C) The expressions of deep-layer CPNs markers are shown in a UMAP visualization of the clusters identified in cells from fetal cortex in mouse. (D) Dot plot of deep-layer CPNs markers expression are shown in mouse projection neuron.

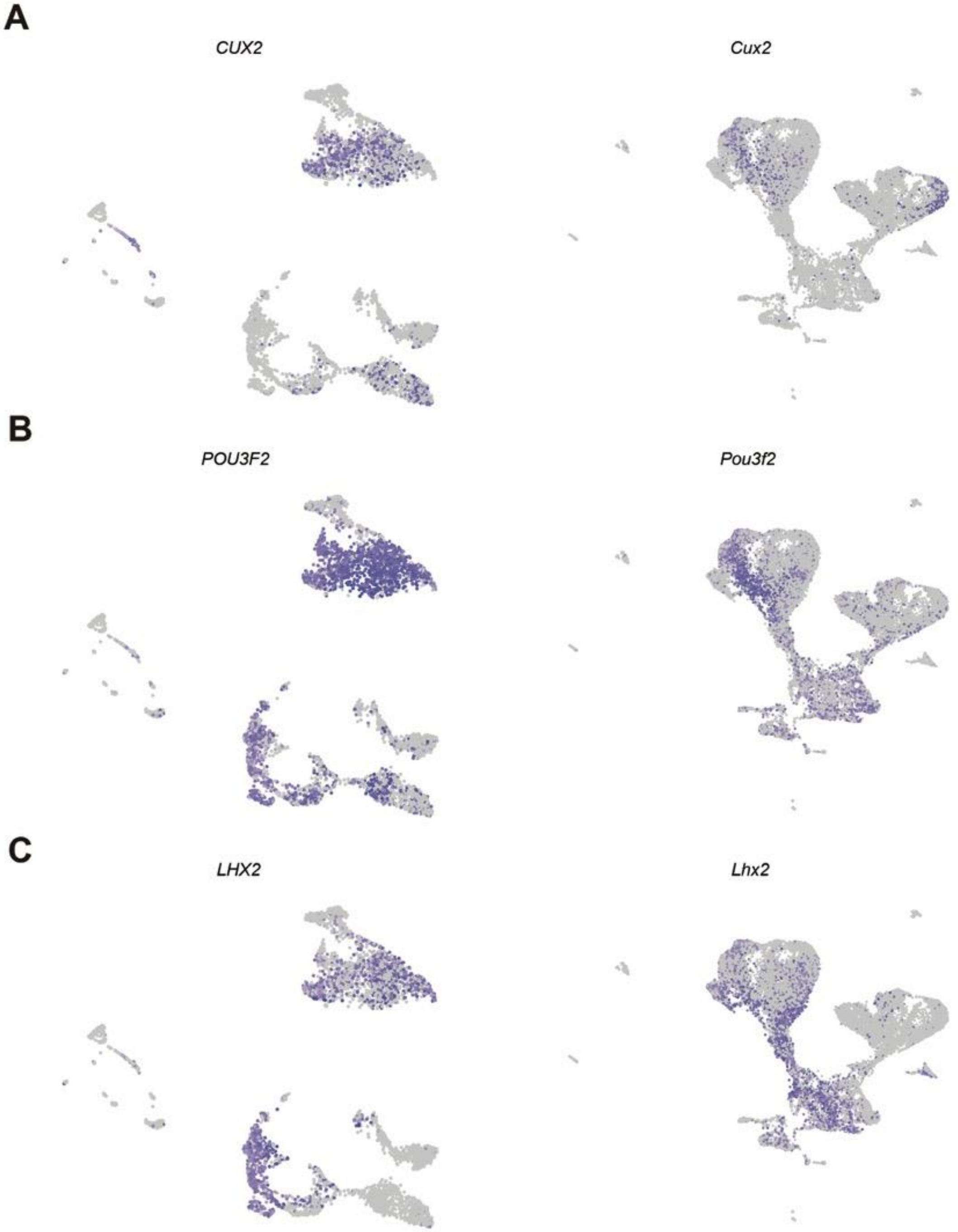
A
CUX2
Cux2
B
POU3F2
Pou3f2
C
LHX2
Lhx2

Supplementary Figure S6. Cell distribution with ULNs marker genes. Cells with (A) *Cux2*, (B) *Lhx2* and (C) *Pou3f2* expression are shown in a UMAP visualization (**Supplementary Figure S2A**) of the clusters identified in cells from fetal cortex in human (left) and mouse (right).

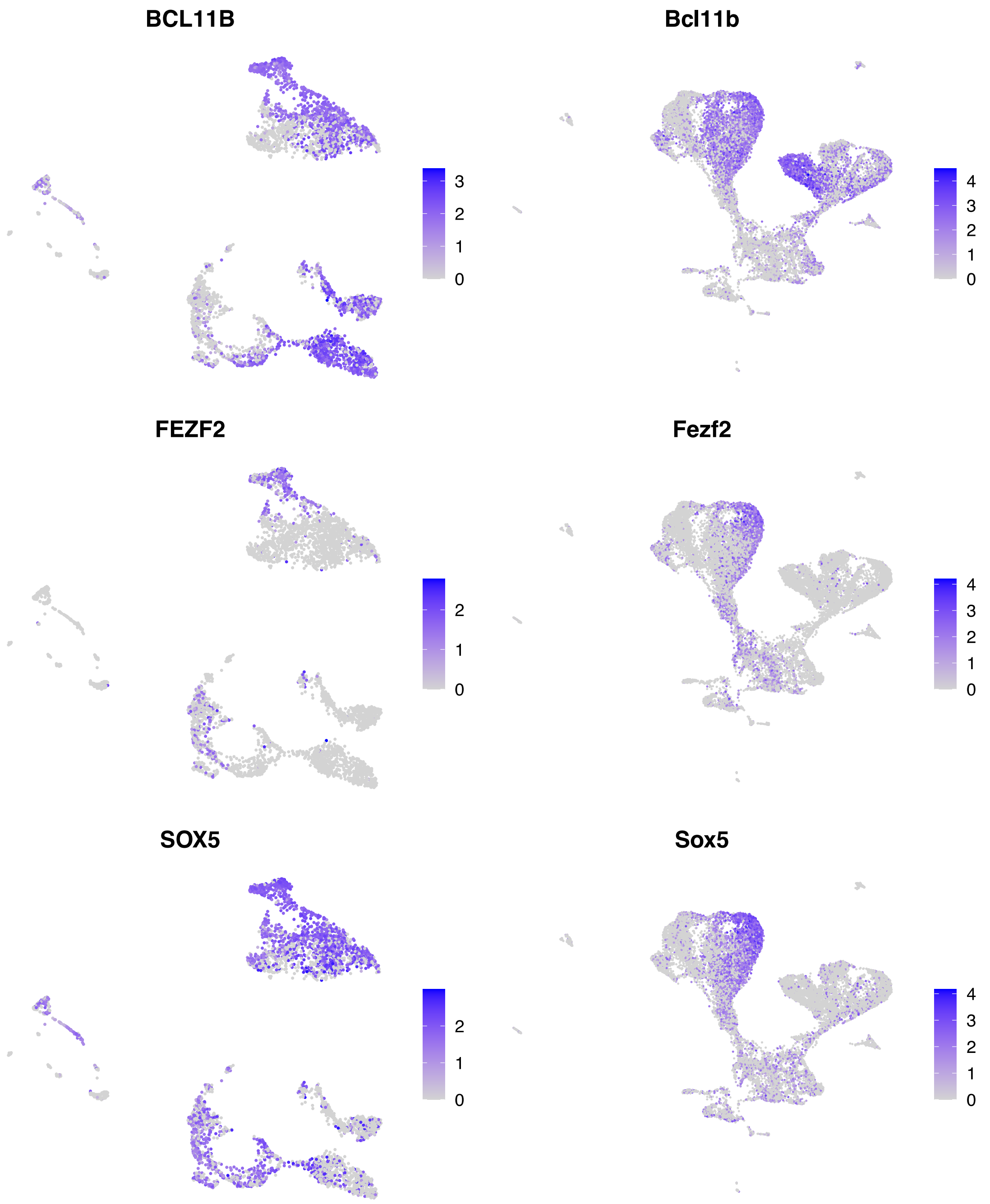

Supplementary Figure S7. Cell distribution with DLNs marker genes. Cells with (A) (A) *Sox5*, (B) *Bcl11b* and (C) *Fezf2* expression are shown in a UMAP visualization (**Supplementary Figure S2A**) of the clusters identified in cells from fetal cortex in human (left) and mouse (right).

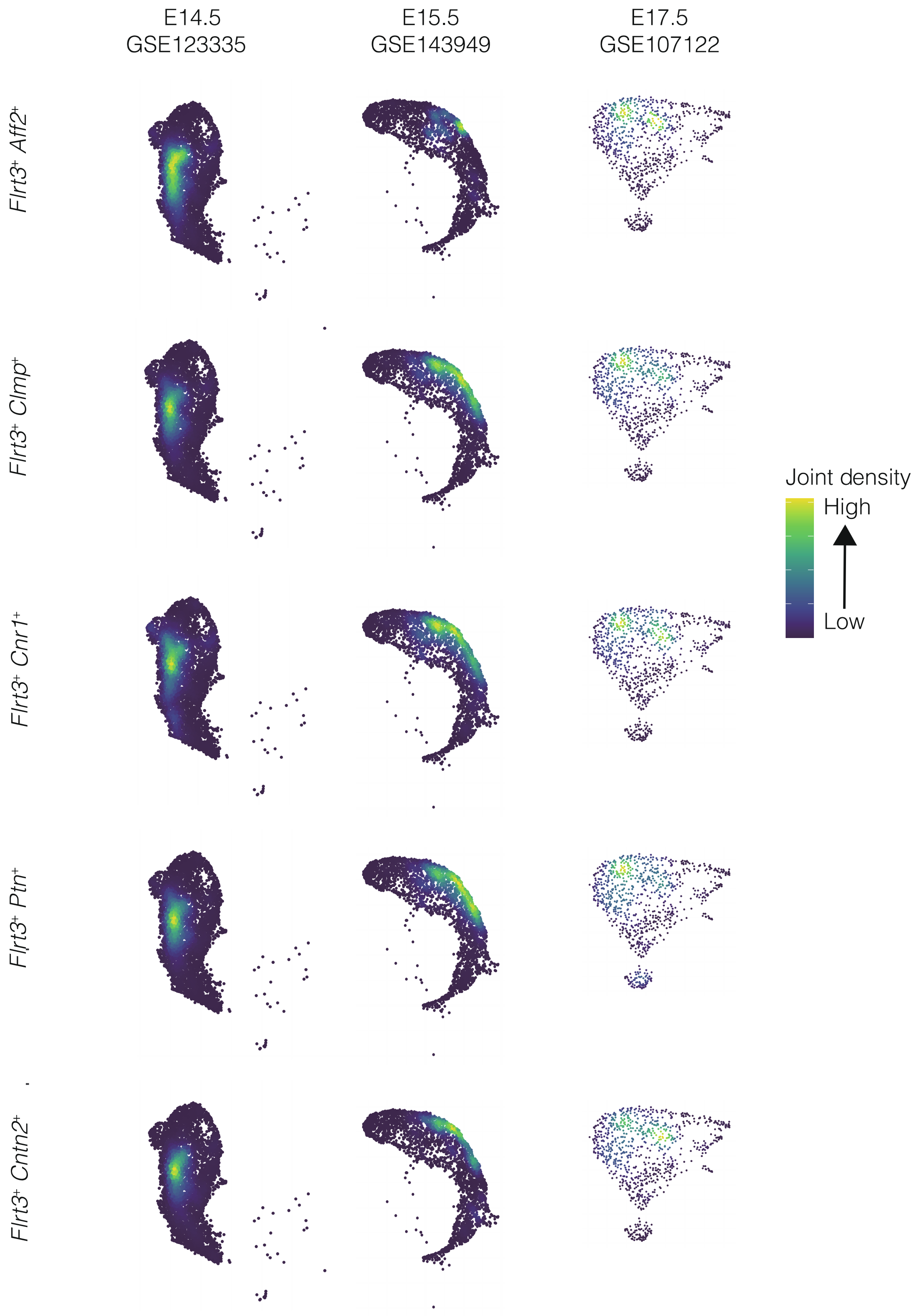
E14.5
GSE123335
E15.5
GSE143949
E17.5
GSE107122
Flrt3+ Aff2+
Flrt3+ Clmp+
Flrt3+ Cnr1+
Flrt3+ Ptn+
Flrt3+ Cntn2+
Joint density
High
Low

Supplementary Figure S8. Co-expression patterns of the six mice D-T-U specific genes from E14.5 to E17.5 mouse cortexes. Cells with *Flrt3*, *Aff2*, *Clmp*, *Cnr1*, *Ptn* and *Cntn2* co-expression identified are shown in each UMAP joint density visualization with data from E14.5, E15.5 and E17.5 mouse cortex. The reference number of each dataset is shown in the upper panel.

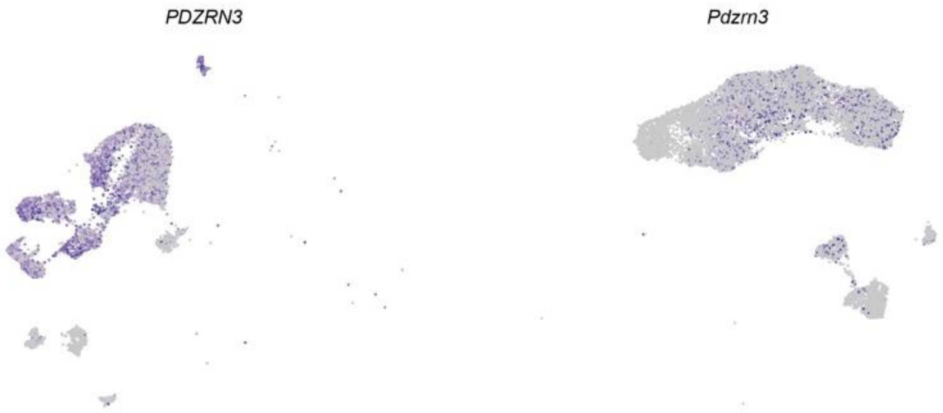

Supplementary Figure S9. Cell distribution with *PDZRN3* in human and mouse adult prefrontal cortex. Cells with *PDZRN3* expression are shown in a UMAP visualization of the clusters identified in cells from fetal cortex in human (left) and mouse (right).
