# Supplementary material for: Single-cell analysis reveals specific neuronal transition during mouse corticogenesis
Source: Front Cell Dev Biol. 2023 Nov 6;11:1209320. doi: 10.3389/fcell.2023.1209320 (PMC10657809; doi:10.3389/fcell.2023.1209320)
Supplement: Supplementary file 2 [file DataSheet1.PDF]

Supplementary Table S1 Data usage for this study

Species	Age	Cells	Area	References
Human	5.85 to 37 gw	4,177	Cerebral Cortex (PFC/V1)	Nowakowski et al
Human	9 to 23 gw	7,262	Cerebral Cortex (FL/PL/OL/TL)	Fan et al
Human	17 to 18 gw	13,284	Cerebral Cortex (Coronal sections)	Polioudakis et al
Human	Adulthood	17,336	Cerebral Cortex (PFC)	Li et al
Mouse	E14.5 and P0*	18,446	Cerebral Cortex	Loo et al
Mouse	E13.5, E15.5 and E17.5*	7,702	Cerebral Cortex	Yuzwa et al
Mouse	E15.5*	5,777	Cerebral Cortex	Li et al
Mouse	E10.5, E12.5, E14.5, E15.5, E16.5, and E18.5*	10,261	Cerebral Cortex	Ruan et al
Mouse	Adulthood	24,810	Cerebral Cortex (PFC)	Bhattacherjee et al
Monkey	110 PCD	10,222	Cerebral Cortex	Zhu et al

*E refers to the embryonic day of the fetal mice, while P refers to the day after post-partum for the postnatal mice.

Supplementary Table S2. Timpoints with sample numbers in human used in this study

Timepoint for sample collection	Number of sample[illegible]
GW5.85	1
GW7	2
GW8	2
GW9	3
GW10	2
GW11	3
GW11.5	1
GW12	4
GW13	5
GW13.3	2
GW14	6
GW14.2/14.5/14.7	5
GW15	2
GW16	3
GW16.3/16.5	2
GW17	5
GW17.3/17.5/17.7	3
GW18	4
GW18.5	2
GW19	3
GW20	4
GW21.5	2
GW22	2
GW23	1
GW24	3
GW25	1
GW26	3
GW32	1
GW37	1
Adult	3

Supplementary Table S3. Timpoints with sample numbers in mouse used in this study

Timepoint for sample collection	Number of samples
E11.5	12
E13.5	16
E14.5	6
E15.5	21
E17.5	8
P0	3
Adult	12

Supplementary Table S4. Marker genes used in this study

Markers	Celltype
HES5	Progenitor
MKI67	Progenitor
GLI3	Progenitor
EOMES	Progenitor
NEUROG2	Progenitor
NEUROD6	ExN
NEUROD2	ExN
BCL11B	DLN
FEZF2	DLN
SOX5	DLN
LHX2	ULN
CUX2	ULN
POU3F2	ULN
LHX6	IN
SST	IN
GAD2	IN
ALDH1L1	Astrocytes
APOD	Astrocytes
BCAN	Astrocytes

Supplementary Table S5. Cells identified in the analysis of dynamic change of cellular compositions

Human

	Progenitor	Astrocyte	DLN	ULN	Interneuron
GW9	302	0	60	0	3
GW10	510	0	514	0	23
GW11	209	1	515	0	67
GW12	771	0	957	4	60
GW13	225	3	150	120	192
GW14	783	4	189	103	153
GW17	6406	1	1168	5224	1444
GW18	5607	1	982	4711	870
GW19	18	1	3	51	47
GW22	8	27	5	48	54
Adult	0	2081	4370	4779	3755

Mouse

	Progenitor	Astrocyte	DLN	ULN	Interneuron
E11.5	1281	0	389	0	0
E13.5	767	0	710	0	146
E14.5	3303	7	3655	17	1783
E15.5	1192	35	2022	353	534
E17.5	201	28	361	457	488
P0	1128	709	405	1760	1655
Adult	0	1540	6554	7979	1126
